# One-pot synthesis of (−)-Ambrox

**DOI:** 10.1038/srep32650

**Published:** 2016-09-01

**Authors:** Shaoxiang Yang, Hongyu Tian, Baoguo Sun, Yongguo Liu, Yanfeng Hao, Yanyu Lv

**Affiliations:** 1 Beijing Innovation Centre of Food Nutrition and Human Health, Beijing Key laboratory of Flavour Chemistry, Beijing Technology and Business University, No. 11 Fucheng Road, Haidian District, Beijing 100048, P.R. China

## Abstract

(−)-Ambrox is recognised as the prototype of all ambergris odorants. Widely used in perfumery, (−)-Ambrox is an important ingredient due to its unique scent and excellent fixative function. An environmentally friendly and practical preparation of (−)-Ambrox is still unavailable at present although a lot of attention has been paid to this hot research topic for many years. A one-pot synthesis of (−)-Ambrox was studied starting from (−)-sclareol through oxidation with hydrogen peroxide in the presence of a quaternary ammonium phosphomolybdate catalyst {[C_5_H_5_NC_16_H_33_] [H_2_PMo_12_O_40_]}, which gave the product a 20% overall yield.

Ambergris is a rare product that is produced in the digestive tract of the sperm whale (*Physeter macrocephalus*) as a waxy substance, presumably to protect it from injuries caused by the sharp beaks of the giant squid (a major dietary staple)^1^. Ambergris is an indispensable ingredient in the manufacture of perfumes as a fixative, for providing the long-term scent, aiding in mixing ingredients and extending the shelf life of perfumes^2^. In the 1930 s Firmenich (the world’s largest privately owned company in the fragrance and flavour business) initiated a vast research program into the composition of ambergris due to its limited supply and relatively high cost^3^. Stoll and his co-workers identified (−)-Ambrox as the most important constituent of the natural ambergris in the 1940 s^4^. Since then (−)-Ambrox has been recognised as the prototype of all ambergris odorants and used as a valuable ingredient in perfumery because of its unique scent and fixative function^5^.

Due to its high price and low availability various synthetic alternatives to ambergris have been developed^6^. Stoll completed the synthesis of Ambrox for the first time by the reduction of sclareolide with LiAlH_4_, followed by an acid-catalysed cyclization of the resulting diol to produce (−)-Ambrox^7^. Several other synthetic routes were reported to prepare Ambrox starting with natural terpenoids^8^,^9^. For commercial syntheses, the major starting materials investigated included homofarnesic acid, homofarnesol, monocyclohomofarnesic acid, sclareol and monocyclohomofarnesol^10^,^11^,^12^,^13^. (−)-Sclareol has been extensively used for preparing (−)-Ambrox due to its reasonably priced commercial availability as extract of *Salvia sclarea* L. All the chemical routes reported to synthesise Ambrox involve several chemical steps having high processing costs, long reaction times, and severe processing conditions such as high pressure and temperature.

The search for alternative cleaner, safer, and environmentally friendly technologies is a priority in the chemistry field^14^. Traditional “stop-and-go” synthesis comprises reaction, workup, and purification^15^. Compared with this traditional approach, a one-pot synthesis is a much more efficient method of achieving several transformations and forming several bonds in one step, while at the same time cutting out several purifications, minimising the generation of waste chemicals and saving time. Thus, an environmentally benign one-pot system should be considered when planning a synthesis^16^. A large range of compounds has been synthesised by one-pot synthesis including Dibenzofurans^17^, Spiropyrazolones^18^, 2,3-Difunctionalized 4-Chlorofurans^19^, (S)-Baclofen^20^, ABT-341^21^ and prostaglandin E1 (PGE1) methyl ester^22^, since this concept was proposed.

In our preliminary research, (−)-Ambrox was detected by accident in 2% yield during the preparation of sclareolide through oxidation of sclareol with hydrogen peroxide catalysed by Na_2_WO_4_·2H_2_O. It became apparent that (−)-Ambrox could be prepared from sclareol using a one-pot reaction if a suitable catalyst was selected. Therefore, in our present work, one-pot synthesis of (−)-Ambrox was investigated starting from (−)-sclareol through oxidation with hydrogen peroxide in the presence of a quaternary ammonium phosphomolybdate catalyst in order to improve the existing preparation methods mentioned above.

## Results and Discussion

**Figure 1:**
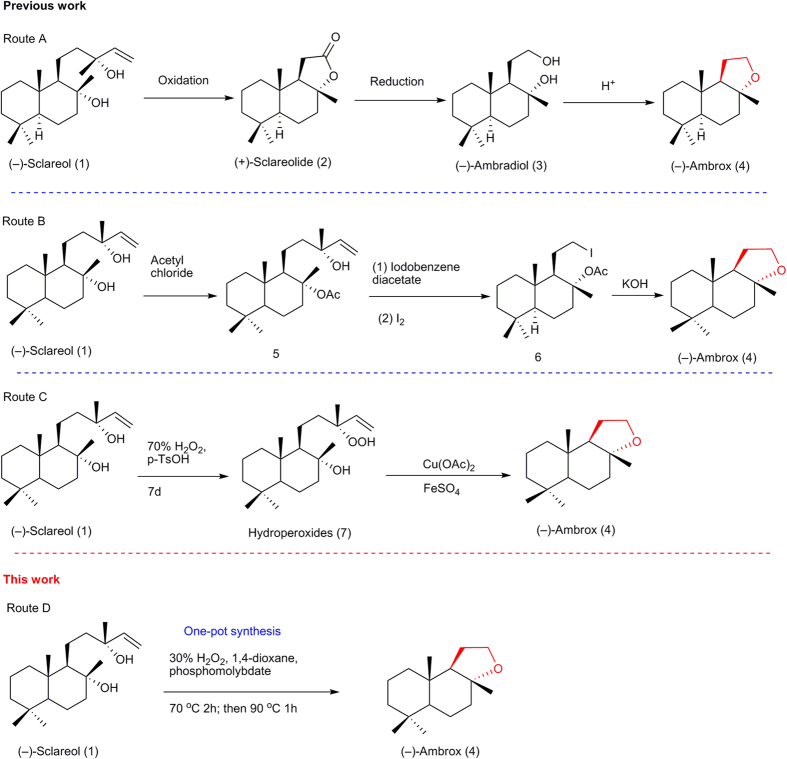
Approaches to (−)-Ambrox.

The results were particularly significant in early research. But chemists were seeking an environmentally friendly and economical method of synthesising (−)-Ambrox. To date, an array of approaches has emerged for the synthesis of (−)-Ambrox with (−)-sclareol as the starting material. The approaches can be divided into three main categories (Fig. 1).

In route A (−)-Ambrox was synthesised through three steps^23^,^24^,^25^, including an oxidative degradation of the (−)-sclareol side chain to give sclareolide, a reduction of sclareolide to ambradiol and a cyclodehydration of ambradiol. It is the most efficient way so far. This system is used in industrial production of (−)-Ambrox in spite of several disadvantages, such as expensive or toxic reagents, elaborate operation, long reaction time and massive pollutant discharge.

In route B alkoxy radicals of several derivatives of sclareol underwent β-fragmentation reaction to provide intermediate 1-(2-iodoethyl)-2,5,5,8a- tetramethyldecahydronaphthalen-2-yl acetate, which was then converted to (−)-Ambrox after three step reactions in 20% overall yield^26^. Stoichiometric amounts of iodobenzene diacetate and iodine were required, which might result in serious pollution and corrosion of equipment.

In route C (−)-sclareol was transformed into (−)-Ambrox through two steps via β-cleavage of an alkoxy radical intermediate in 11–12% overall yield^27^. The oxidation was carried out using 70% H_2_O_2_ as the oxidant in the presence of stoichiometric amounts of Cu(OAc)_2_·2H_2_O, FeSO_4_·7H_2_O for 7 d. Obviously this process is not practical due to its low efficacy, possible safety risks and large pollutant discharge.

In our preliminary experiment, (−)-sclareol was oxidized by H_2_O_2_ in the presence of Na_2_WO_4_·2H_2_O (10 mol %) and NaH_2_PO_4_ (0.1 equiv) as the catalyst, and TBAB as phase transfer catalyst in 1,4-dioxane at 70 °C for 2 h to give 2% yield of (−)-Ambrox. It was interesting to learn that if a suitable catalyst was selected, (−)-Ambrox could be prepared by one-pot reaction. The oxidant is hydrogen peroxide, which is a green oxidant. It is probably the best terminal oxidant after dioxygen with respect to environmental and economic considerations^28^.

In order to improve the yield of (−)-Ambrox, the reaction mixture was heated to 90 °C after stirring for 2 h at 70 °C. The effect of reaction time at 90 °C on yield was explored and the results are shown in Table 1. The yield of (−)-Ambrox reached the highest value of 3.86% after 60 min (Entry 7, Table 1) and decreased gradually with extended reaction times. Obviously, this was a promising but not ideal yield. It was predicted that various phosphotungstate catalysts might be more effective in view of the real catalytic species produced by the reaction of Na_2_WO_4_·2H_2_O with NaH_2_PO_4._

Phosphotungstate catalyst is one of the Polyoxometalates (POMs). POMs are discrete metal-oxide clusters of W, Mo, V, and Nb that have been attracting increasing interest because of their multi-electronic redox activities, photochemical properties, acidic properties and magnetic properties, resulting in potential applications of POMs as catalysts and functional materials^29^. The Ishii-Venturello system has been used on the epoxidation reaction successfully^30^,^31^. As Mo and W belong to the same main group, they display similar characteristics. So our main object is to look for effective phosphotungstate and phosphomolybdate catalysts. A series of quaternary ammonium phosphotungstates and phosphomolybdates were prepared^30^ and listed in Table 2. All these self-made catalysts were tried as a way of catalysing the oxidation of (−)-sclareol with H_2_O_2_ in 1,4-dioxane at 70 °C for 2 h, and then at 90 °C for 1 h. The results are given in Table 3. The catalyst 10 p gave the best yield of 18.20% (entry 20, Table 3), which was almost five times as much as that given by Na_2_WO_4_·2H_2_O. The product was isolated and purified easily. The quaternary ammonium phosphomolybdates usually displayed better catalytic ability (entries 16–21, Table 3) than quaternary ammonium phosphotungstates (entries 4–15, Table 3). All the quaternary ammonium phosphotungstates belong to Keggin-type phosphotungstates^30^. To explore the catalytic capability of different types of phosphotungstates Dowson-type phosphotungstate HPC (H_6_P_2_W_18_O_62_.H_2_O) was also prepared^32^. The yield given by HPC was 5.47%, which was very close to those values produced by Keggin-type phosphotungstates. The results in Table 3 show that the optimum catalyst for one-pot synthesis for (−)-Ambrox is compound 10 p {[C_5_H_5_NC_16_H_33_] [H_2_PMo_12_O_40_]}.

The amount of catalyst 10 p for the reaction was optimised (entries 1–10, Table 4). (−)-Ambrox was produced in the highest yield of 22.79% when 3% equiv. catalyst was used (entry 3, Table 4). The yield decreased from 22.79% to 16.58% when the catalyst loading was lowered from 3% to 1%, whereas the yield didn’t increase with the increment of catalyst loading when it exceeded 3%.

The reaction time was also optimised when the reaction was catalysed by 10 p. The results are shown in Table 4 (entries 11–20). The results indicate that the yield of (−)-Ambrox increased gradually with extended reaction times from 0 min to 60 min at 90 °C (entries 11–17, Table 4), but decreased from 60 min to 90 min (entries 17–20, Table 4). (−)-Ambrox was obtained in the highest yield of 22.17% after 60 min at 90 °C, which was similar to the case catalysed by Na_2_WO_4_·2H_2_O.

The reaction was repeated under the above optimised conditions and (−)-Ambrox was obtained in an average isolated yield of 20%. The determination of its specific rotation was [α] = −30° (C = 1, toluene), which was similar to that in other literature^24^.

**Figure 2:**
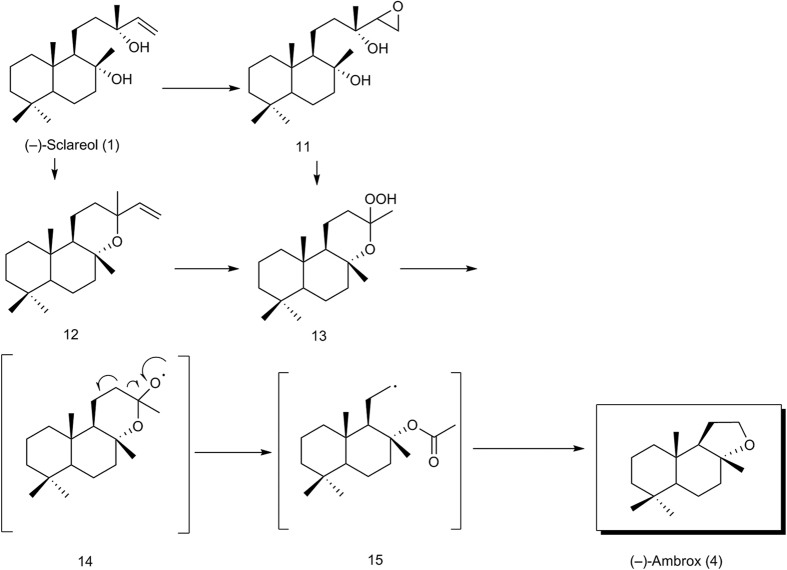
Proposed one-pot synthesis reaction mechanisms.

The plausible one-pot synthesis reaction mechanisms were proposed for the current protocol (Fig. 2). (−)-Sclareol was initially epoxidised to compound 11 (Figure S5) or converted to compound 12 (Figure S6) by intramolecular etherification in the presence of the quaternary ammonium phosphomolybdate 10 p. Subsequently, compound 11 or 12 might undergo catalysed substitution with hydrogen peroxide to produce intermediate 13, which fragmented by radical mechanism through radical intermediates 14 and 15 to produce (−)-Ambrox. This one-pot synthesis proceeds through epoxidation^30^, intramolecular etherification^33^, free radical substitution^27^ and free radical fragmentation^26^.

A one-pot synthesis of (−)-Ambrox has been accomplished by the use of (−)-sclareol, thus demonstrating the power of catalysis in the synthesis of natural products. This synthesis was made possible by the discovery of a novel route to preparing (−)-Ambrox.

## Conclusions

In summary, total syntheses of (−)-Ambrox has been accomplished in 20% total yield by using an inexpensive and simple catalyst and one-pot reactions. This route is not only short and efficient but also has several noteworthy and sustainable features: (1) The total synthesis is performed in only one isolation and chromatographic purification, which reduces the amount of solvent needed and waste formed. (2) The reaction is a highly selective catalytic reaction, involving a quaternary ammonium phosphomolybdates catalyst with 3% catalyst loading and reduces the generation of waste. (3) The oxidant employed in the present synthesis is hydrogen peroxide and water without any pollution is the only theoretical by-product. Thus, the present one-pot synthesis is not only efficient for the synthesis of (−)-Ambrox, but is also environmentally benign. This work is significant in improving the synthesis of (−)-Ambrox based on (−)-sclareol. We are still doing research in our lab in order to further improve the yield of (−)-Ambrox.

## Methods

### Synthesis of the catalysts

Synthesis of catalysts was illustrated by the synthesis of catalyst 10 p.

PMA (1.82 g, 1 mmol) and deionized water (10 mL) were combined in a 50 mL three-neck flask. The mixture was stirred for 5 min at 25 °C and further CPC (0.36 g, 1 mmol) in deionized water (10 mL) was added after 5 min, then the mixture was stirred for 3 h at 25 °C. When filtered, the filtrate cake was washed with liquid and dried by vacuum to produce H1 (1.76 g, 82%) as a dark green solid.

### One-pot synthesis (−)-Ambrox

One-pot synthesis of (−)-Ambrox was illustrated by the reaction of catalysed 10 p.

Sclareol (3.08 g, 10 mmol), 10 p (0.65 g, 0.3 mmol, 3% equiv), 30%H_2_O_2_ (5 mL), 1, 4-dioxane (20 mL) were combined in a 50 mL three-neck flask. The mixture was stirred for 2 h at 70 °C and then 1 h at 90 °C. The 1, 4-dioxane was removed under reduced pressure. The residue was extracted with ethyl acetate (20 mL × 3), the organic phases were then washed with a saturated solution of Na_2_CO_3_ and brine and then dried over MgSO_4_. After solvent removal, the residue was purified by flash chromatography on silica gel (petroleum/EtOAc, 30:1) to produce (−)-Ambrox (0.47 g, 20%) as a colourless solid m.p., 74–75 °C, 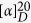 = −30° (C = 1, toluene).

^1^H NMR (300 MHz, CDCl_3_) δ: 0.81–0.89 (9H, m, Me-10, 2Me-4), 1.08(3H, s, Me-8), 1.94 (1H, d, *J* = 11.1 Hz, H-11), 3.82 (1H, q, *J* = 8.0 Hz, H-12), 3.91 (1H, q, *J* = 6.8 Hz, H-12) (Figure S2).

^13^C NMR (75 MHz, CDCl_3_) δ: 15.0(C-20), 18.4(C-2), 20.6(C-6), 21.1(C-19), 22.6(C-17), 22.6(C-11), 33.0(C-4), 33.6(C-18), 36.1(C-10), 39.7(C-7), 39.9(C-1), 42.4(C-3), 57.2(C-5), 6 0.1(C-9), 64.9(C-12), 79.9(C-8) (Figure S3).

IR (KBr) ν:3751, 2920, 2869, 1457, 1382, 1273, 1200, 1125, 1005, 914, 839, 715, 478, 416 cm^−1^ (Figure S4).

## Additional Information

**How to cite this article**: Yang, S. *et al*. One-pot synthesis of (−)-Ambrox. *Sci. Rep.* **6**, 32650; doi: 10.1038/srep32650 (2016).

## Supplementary Material

Supplementary Information

## Figures and Tables

**Table 1:** Optimization of the Reaction Times.

Entry	Reaction time	4 Yield (%)	Entry	Reaction time	4 yield (%)	
1	0 min	2.00	11	2 h	2.95	
2	10 min	2.46	12	3 h	3.00	
3	20 min	2.77	13	4 h	3.01	
4	30 min	2.87	14	5 h	2.74	
5	40 min	3.35	15	6 h	2.67	
6	50 min	3.42	16	7 h	2.63	
7	60 min	3.86	17	8 h	2.54	
8	70 min	3.64	18	9 h	2.46	
9	80 min	3.53	19	10 h	2.38	
10	90 min	3.20	20	11 h	2.10	

*Reaction conditions: (−)-Sclareol 1 (10 mmol), Na_2_WO_4_·2H_2_O (0.1 equiv), NaH_2_PO_4_ (0.1 equiv), TBAB (0.1 equiv), 1, 4-dioxane (7 mL), H_2_O_2_ (5 mL), 70 °C, 2 h; then rising to 90 °C; TBAB (Tetrabutyl ammonium bromide); GC yield.

Reaction time starts counting when the reaction system temperature rises to 90 °C.

**Table 2:** Synthesis of the catalysts.

Entry	8	9	8:9	10	Chemical compositions of catalyst	Yield (%)
1	PTA	HTAC	1:1	10a	{[(CH_3_) _3_C_16_H_33_N] [H_2_PW_12_O_40_]}	80
2	PTA	HTAC	1:2	10b	{[(CH_3_) _3_C_16_H_33_N]_2_ [HPW_12_O_40_]}	78
3	PTA	HTAC	1:3	10c	{[(CH_3_) _3_C_16_H_33_N]_3_ [PW_12_O_40_]}	81
4	PTA	CPC	1:1	10d	{[C_5_H_5_NC_16_H_33_] [H_2_PW_12_O_40_]}	76
5	PTA	CPC	1:2	10e	{[C_5_H_5_NC_16_H_33_]_2_ [HPW_12_O_40_]}	80
6	PTA	CPC	1:3	10f	{[C_5_H_5_NC_16_H_33_]_3_ [PW_12_O_40_]}	75
7	PTA	HMAC	1:1	10g	{[(CH_3_) _4_N] [H_2_PW_12_O_40_]}	88
8	PTA	HMAC	1:2	10h	{[(CH_3_) _4_N]_2_ [HPW_12_O_40_]}	82
9	PTA	HMAC	1:3	10i	{[(CH_3_) _4_N]_3_ [PW_12_O_40_]}	82
10	PTA	TBAB	1:1	10j	{[(CH_3_CH_2_ CH_2_ CH_2_)_4_N] [H_2_PW_12_O_40_]}	83
11	PTA	TBAB	1:2	10k	{[(CH_3_CH_2_ CH_2_ CH_2_)_4_N]_2_ [HPW_12_O_40_]}	77
12	PTA	TBAB	1:3	10l	{[(CH_3_CH_2_ CH_2_ CH_2_)_4_N]_3_ [PW_12_O_40_]}	79
13	PMA	HTAC	1:1	10m	{[(CH_3_) _3_C_16_H_33_N] [H_2_P Mo_12_O_40_]}	72
14	PMA	HMAC	1:1	10n	{[(CH_3_) _4_N] [H_2_P Mo_12_O_40_]}	76
15	PMA	TBAB	1:1	10o	{[(CH_3_CH_2_ CH_2_ CH_2_)_4_N] [H_2_P Mo_12_O_40_]}	70
16	PMA	CPC	1:1	10p	{[C_5_H_5_NC_16_H_33_] [H_2_PMo_12_O_40_]}	82
17	PMA	CPC	1:2	10q	{[C_5_H_5_NC_16_H_33_]_2_ [HPMo_12_O_40_]}	80
18	PMA	CPC	1:3	10r	{[C_5_H_5_NC_16_H_33_]_3_ [PMo_12_O_40_]}	81

*Reaction conditions: 8 (1 mmol), 9 (relative equiv), deionized water (20 mL), 25 °C, 3 h.

PTA (H_3_P W_12_O_40_), PMA (H_3_P Mo_12_O_40_), HTAC (N-Hexadecyltrimethylammonium Chloride), CPC (Cetylpyridinium chloride), HMAC (Tetramethylammonium chloride), TBAB (Tetrabutyl ammonium bromide).

**Table 3:** Optimisation of the catalysts.

Entry	Catalyst	4 yield (%)	Entry	Catalyst	4 yield (%)	
						
1	PTA	6.34	12	10i	1.79	
2	PMA	7.05	13	10j	2.13	
3	HPC	5.47	14	10k	1.47	
4	10a	7.77	15	10l	4.54	
5	10b	4.78	16	10m	10.40	
6	10c	2.03	17	10n	12.30	
7	10d	8.03	18	10o	14.11	
8	10e	5.07	19	10p	18.20	
9	10f	1.98	20	10q	16.78	
10	10g	5.47	21	10r	11.79	
11	10h	6.13				

*Reaction conditions: 1 (10 mmol), catalyst (0.1 equiv), 1,4-dioxane (7 mL), H_2_O_2_ (5 mL), 70 °C, 2 h; then 90 °C, 1h; GC yield; HPC (Dowson-type H_6_P_2_W_18_O_62_).

**Table 4:** Optimisation of the Reaction Conditions when catalysed by 10p.

Entry	Catalyst amount (mmol)*	4 Yield (%)	Entry	Reaction time**	4 Yield (%)	
1	0.01	16.58	11	0	3.40	
2	0.02	19.76	12	10 min	6.87	
3	0.03	22.79	13	20 min	9.91	
4	0.04	20.14	14	30 min	13.99	
5	0.05	20.17	15	40 min	15.38	
6	0.10	19.65	16	50 min	20.78	
7	0.50	19.54	17	60 min	22.15	
8	1.00	18.34	18	70 min	19.98	
9	1.50	18.20	19	80 min	16.20	
10	2.00	17.97	20	90 min	14.25	

*Reaction conditions: (−)-Sclareol 1 (10 mmol), catalyst 10 p (relative equiv), 1,4-dioxane (7 mL), H_2_O_2_ (5 mL), 70 °C, 2 h; then 90 °C, 1h; GC yield.

**Reaction conditions: 1 (10 mmol), catalyst 10 p (0.03 equiv), 1, 4-dioxane (7 mL), H_2_O_2_ (5 mL), 70 °C, 2 h; then 90 °C. Reaction time starts counting when the reaction system temperature rises to 90 °C.
