# Supplementary material for: One-pot synthesis of (−)-Ambrox
Source: Sci Rep. 2016 Sep 1;6:32650. doi: 10.1038/srep32650 (PMC5007644; doi:10.1038/srep32650)

**One-pot synthesis of (−)-Ambrox**

**Shaoxiang Yang*, Hongyu Tian, Baoguo Sun, Yongguo Liu, Yanfeng Hao and Yanyu Lv**

*Beijing Innovation Centre of Food Nutrition and Human Health, Beijing Key laboratory of Flavour Chemistry, Beijing Technology and Business University, No.11 Fucheng Road, Haidian District, Beijing 100048, P.R. China.*

| **TABLE OF CONTENTS** | **PAGE** |
| --- | --- |
| **S1. GC Analysis of (−)-Ambrox** | **2** |
| **S2. ^1^H NMR Spectra of (−)-Ambrox** | **3** |
| **S3. ^13^C NMR Spectra of (−)-Ambrox** | **4** |
| **S4. IR Spectra of (−)-Ambrox** | **5** |
| **S5. MS Spectra of compound 11** | **6** |
| **S6. MS Spectra of compound 12** | **7** |

**S1. GC Analysis** **of (−)-Ambrox**

An Agilent 6890 GC with a flame ionization detector (FID) was used for GC analyses (Agilent Technologies, Santa Clara, CA, USA). The column used DB-WAX(30.0m×250μm，0.25μm). The analytical condition was as follows: injector temperature 240°C, detector temperature 250°C, He as carrier gas, constant flow mode 20 mL/min, split ratio 20/1. The oven temperature was programmed from 140°C to 240°C at a rate of 10°C/min, and held at 240°C for 10 min. The concentration of samples was about 0.5 wt.% in dry ether, the injection volume was about 0.4 μl.

**GC Spectra**

**S2. ^1^H NMR Spectra of (−)-Ambrox**


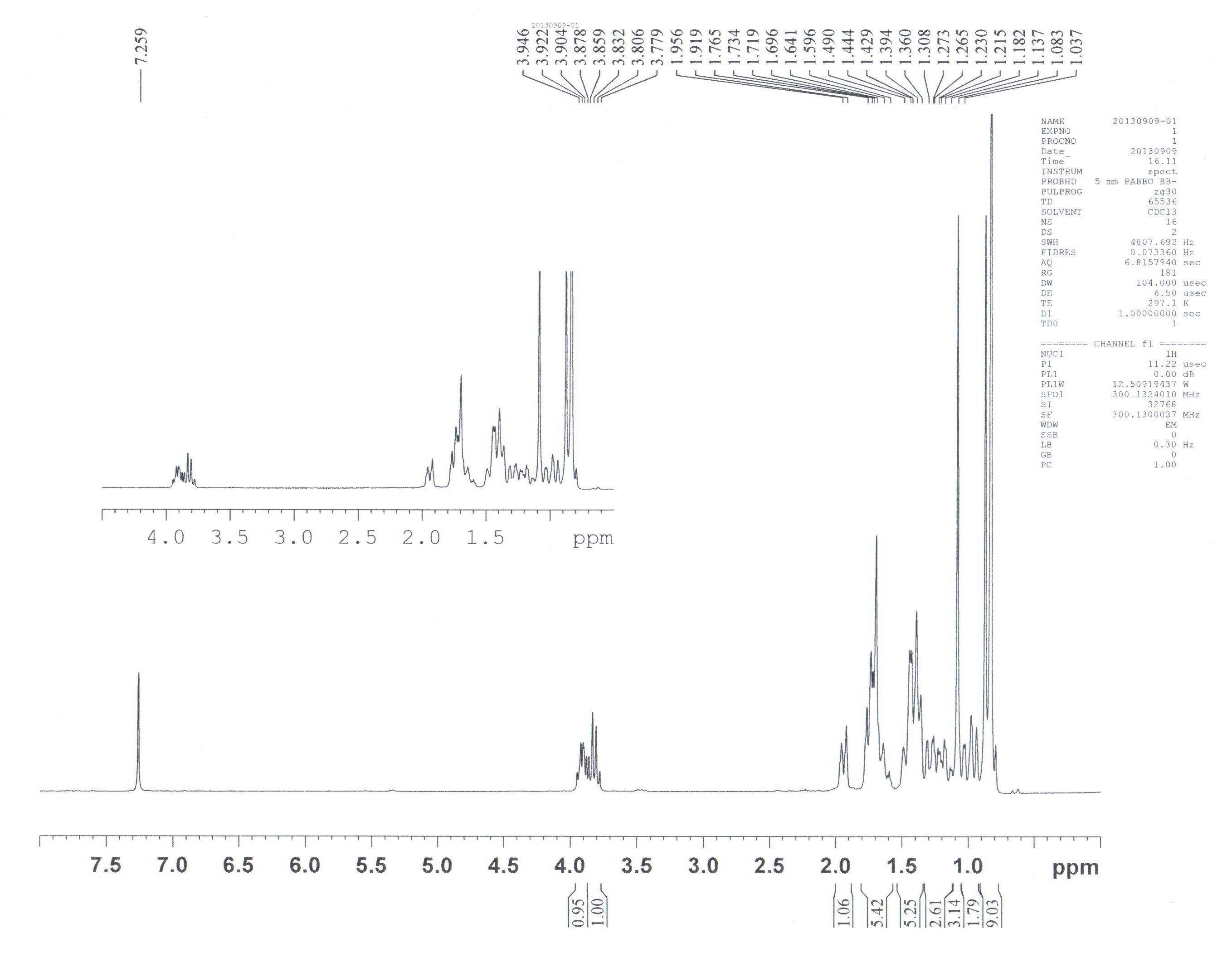


**S3. ^13^C NMR Spectra of (−)-Ambrox**


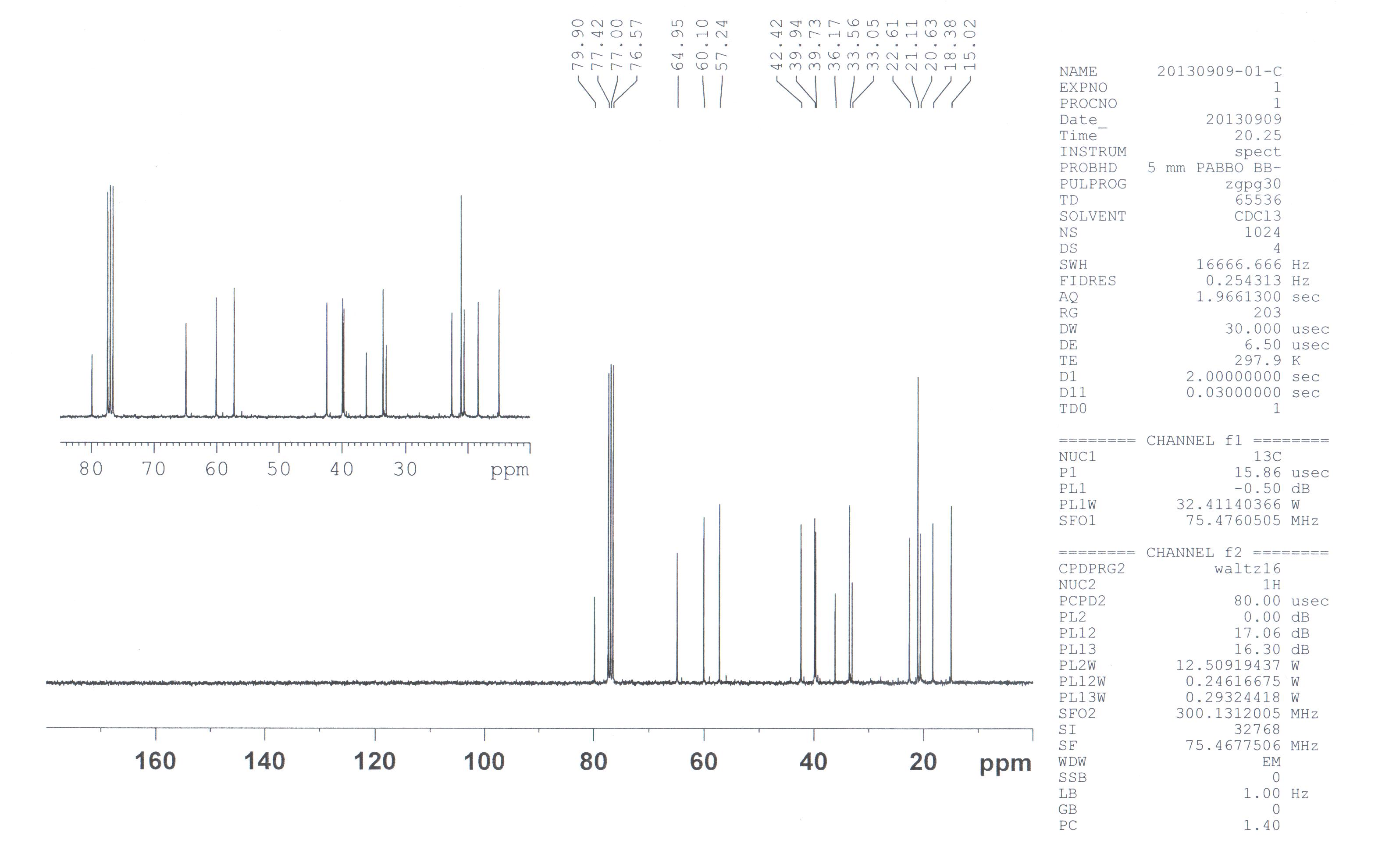


**S4. IR** **Spectra of (−)-Ambrox**

**S5. MS** **Spectra of compound 11**


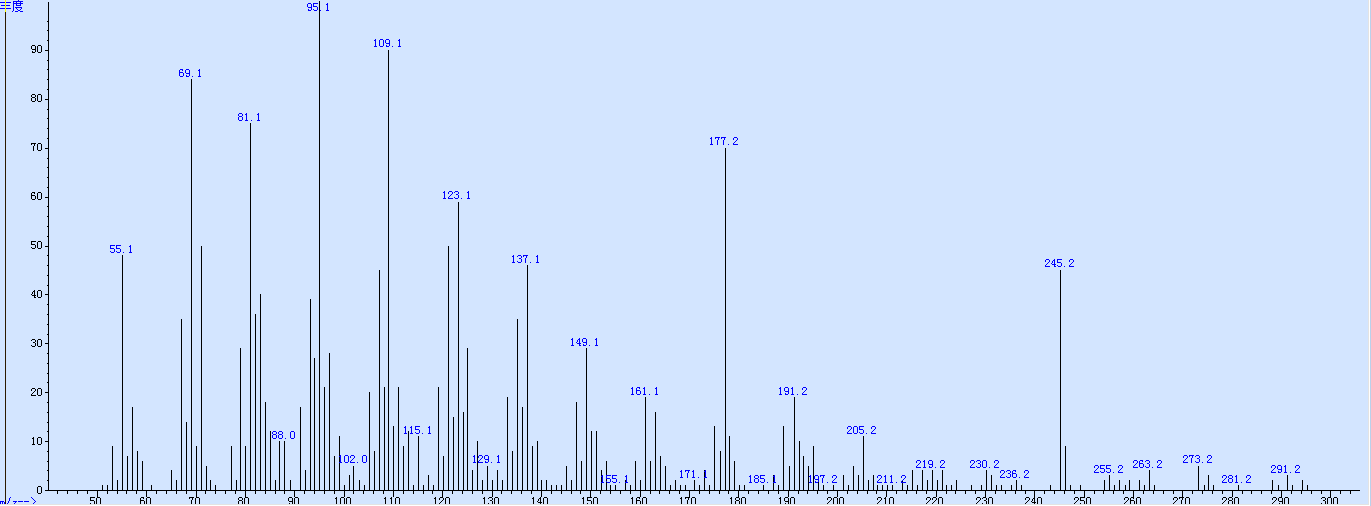


**S6. MS** **Spectra of compound 12**


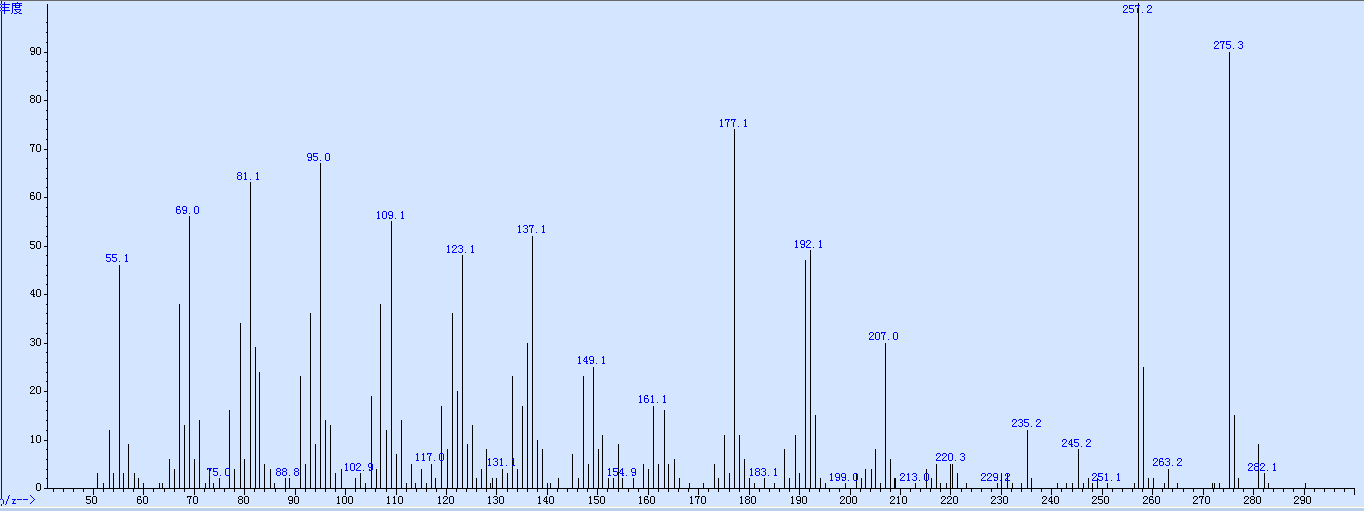

Supplement: Supplementary Information [file srep32650-s1.doc]
